# Folate‐Associated DNA Methylation and Chemotherapy‐Induced Toxicities in Patients With Colorectal Cancer

**DOI:** 10.1002/mnfr.70127

**Published:** 2025-05-27

**Authors:** Nienke R. K. Zwart, Dieuwertje E. Kok, Sajini N. H. Ariyaratne, Jill A. McKay

**Affiliations:** ^1^ Division of Human Nutrition and Health Wageningen University & Research Wageningen The Netherlands; ^2^ Department of Applied Sciences Northumbria University Newcastle upon Tyne UK

**Keywords:** chemotherapy, colorectal cancer, DNA methylation, folate, nutrition, toxicities

## Abstract

Patients with stage II‐III colorectal cancer (CRC) often receive fluoropyrimidine‐based chemotherapy, usually combined with other regimens, of which ∼50% experience severe chemotherapy‐induced toxicities. The B‐vitamin folate has been associated with toxicity risk, possibly through effects on DNA methylation. Here, we examined the potential role of folate‐associated DNA methylation in the context of chemotherapy‐induced toxicities. Systematic literature searches were conducted to identify studies investigating either DNA methylation profiles associated with folate status/intake or with toxicities. Overlapping CpG sites and genes across studies investigating associations for “folate‐DNA methylation” and “DNA methylation‐toxicities” were identified. The probability of overlap was tested using hypergeometric tests and Gene Ontology and KEGG pathway analyses were performed. Six studies were included. A significant number of CpGs and genes overlapped with altered methylation in response to both folate and hand‐foot syndrome (HFS) or thrombocytopenia. Moreover, methylation of genes within the KEGG pathway “focal adhesion” was related to folate status/intake and occurrence of HFS, thrombocytopenia, and neutropenia. We identified some overlapping DNA methylation profiles related to both folate exposures and toxicities. This provides preliminary evidence implying folate‐associated DNA methylation may determine risk of toxicities, and therefore may be considered a modifiable factor for improving patient outcomes.

## Graphical abstract

Over half of patients with colorectal cancer experience severe chemotherapy‐induced side effects. The B‐vitamin folate may play a role in these side effects, potentially by altering DNA methylation, a mechanism of gene regulation. Our comprehensive literature search identified studies investigating DNA methylation associated with folate status/intake or with side effects and observed overlapping DNA methylation patterns. This study provides preliminary evidence suggesting folate‐associated DNA methylation may be linked to chemotherapy‐induced side effects.

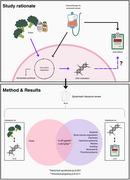

**Abbreviations**
5‐FU: 5‐fluorouracil
CRC: colorectal cancer
DMP: differently methylated positions
DMR: differently methylated regions
GO: gene ontology
HFS: hand‐foot syndrome
KEGG: Kyoto encyclopedia of genes and genomes
ROS: reactive oxygen species

## 1 Introduction

Colorectal cancer (CRC) is the third most commonly diagnosed cancer [1]. Patients with high‐risk stage II and stage III CRC often receive chemotherapy as part of their anti‐cancer treatment [2, 3]. A common chemotherapeutic strategy for CRC consists of a fluoropyrimidine regimen, often 5‐fluorouracil (5‐FU) or capecitabine, with or without oxaliplatin [3]. A substantial number of patients with CRC experience fluoropyrimidine‐induced toxicities, such as diarrhea (∼25%–50%) [4, 5, 6], hand‐foot syndrome (∼25%–60%) [4, 5, 6, 7, 8], and neutropenia (∼5%–20%) [4, 5].

Fluoropyrimidine regimens target the one‐carbon metabolism in which the B‐vitamin folate plays a crucial role [9]. The one‐carbon metabolism is a complex series of biochemical reactions providing nucleotides for DNA synthesis and repair and methyl groups for methylation reactions [10]. Relatively high levels of circulating folate and detectable levels of folic acid in blood have been associated with an increased risk of fluoropyrimidine‐induced toxicities [11, 12, 13]. DNA methylation, referring to the addition of a methyl group to CpG sites [14], is an essential regulator of gene transcription [15] and hence may also play a role in chemotherapy‐induced toxicities [16]. Epigenome‐wide association studies reported that folate intake, both dietary and supplemental in the form of folic acid, was associated with DNA methylation patterns [17, 18, 19, 20].

Deciphering the role of folate‐associated DNA methylation in chemotherapy‐induced toxicities has the potential to improve understanding of the mechanisms behind interindividual differences in toxicities. This will potentially provide a basis to refine treatment strategies and minimize toxicities in patients with CRC and subsequently improving quality of life and prognostic outcomes [21, 22]. Here, we aimed to examine the potential role of folate‐associated DNA methylation in the context of chemotherapy‐induced toxicities.

## 2 Method

Two separate searches were conducted to provide a comprehensive overview of the current evidence available on the relationship between folate/folic acid and DNA methylation in adults (Search 1) and DNA methylation and fluoropyrimidine‐induced toxicities in patients with CRC (Search 2). Data were extracted from publications identified from each search to examine the overlap in methylated CpG sites and genes in relation to both folate/folic acid and chemotherapy‐induced toxicities.

### 2.1 Identification of Studies

Both literature searches were conducted in PubMed on May 30, 2024. Medical Subjects Headings and free text words based on titles, abstracts, and/or keywords were used to search for eligible publications. The search strings are presented in Supporting Information 1. This review focused on human observational and intervention studies. Reference lists of included articles were searched for additional studies. Moreover, narrative or systematic reviews were utilized to identify primary investigations of interest.

For studies focusing folate or folic acid exposures in relation to DNA methylation profiles (Search 1), the study population of interest was the general population (>18 years). The exposure of interest included dietary or supplemental intake as well as circulating biomarkers of folate or folic acid. Studies that did not examine folate or folic acid as exposure variables were excluded. Moreover, circulating levels of homocysteine were not considered an exposure of interest, as homocysteine levels do not only reflect folate status but also that of other B‐vitamins [23]. The outcome of interest was genome‐wide DNA methylation measured in blood by either the Illumina Infinium HumanMethylation450k Bead Chip (450K) array or the Infinium MethylationEPIC BeadChip (EPIC) array.

For studies focusing on DNA methylation profiles in relation to chemotherapy‐induced toxicities (Search 2), the study population of interest was adults with CRC receiving fluoropyrimidine‐based chemotherapy. The exposure of interest was genome‐wide DNA methylation measured in blood or tissue by either the 450K array or the EPIC array. The outcome of interest was any toxicity or measures of toxicities (e.g., blood cell count), which referred to acute or chronic adverse events of the chemotherapy. The toxicities could be reported by health care professionals or patients and measured objectively or subjectively.

After removal of duplicates, the titles, abstracts, and full text articles were evaluated by two authors (NRKZ and SNHA or JAM). Publications were excluded if the studies were (1) classified as animal experiments or in vitro studies; (2) not among the general population (>18 years) (Search 1) or not among adults with CRC receiving fluoropyrimidine‐based chemotherapy (Search 2); (3) not investigating the exposure or outcome of interest as described above separately per search; (4) not containing (full) original data; (6) non‐English; and (7) published before 2008 (the year the 450K array was released).

### 2.2 Data Integration

Following the literature searches, data integration was undertaken to explore the overlapping DNA methylation profiles associated with folate exposures and chemotherapy‐induced toxicities. For each included study, CpG sites and gene lists were compiled. The CpGs were determined as CpGs that were associated with either folate exposures or toxicities at the CpG‐specific level, including CpGs in differently methylated regions (DMRs) that were related to folate exposures or toxicities. The DMRs were mapped to their corresponding CpGs using the GenomicRanges [24] and IlluminaHumanMethylation450kanno.ilmn12.hg19 package [25] in R‐studio (version 4.1.0), which could result in redundant extracted CpGs from DMRs as the excluded probes due to non‐CpG sites, low quality, and so forth, were not known for the individual studies. However, compared to the number of CpGs mentioned in the publications, the redundantly extracted CpGs from DMRs were minimal (<10%) (Supporting Information 2). The genes list included gene symbols corresponding to individual CpGs (differently methylated positions, DMP) or DMRs.

### 2.3 Comparison of Methylation Profiles

We performed a comparative analysis of the CpG and gene lists, using CpG IDs and gene symbols as the common identifiers. The probability of the observed overlap in DNA methylation profiles, both at CpG and gene level, was evaluated using hypergeometric tests. The population size for tests on CpG level was determined by the lowest number of CpG sites measured for methylation status by the arrays used in each study (450K: 431,312; EPIC: 753,722). For the tests on the gene level, the population size was set at the lowest number of RefSeq gene symbol identifiers on the arrays used in each study (450K: 21,231; EPIC: 27,364). A *p* value below 0.05 was deemed statistically significant.

### 2.4 GO and KEGG Pathway Analyses

The Database for Annotation, Visualization, and Integrated Discovery (DAVID) [26] was employed to perform Gene Ontology (GO) and Kyoto Encyclopedia of Genes and Genomes (KEGG) pathway analyses (June 2024). The GO and KEGG analyses were conducted individually for each of the included studies. GO processes with a Benjamini‐Hochberg *p* value < 0.05 and KEGG pathways with standard *p* value < 0.05 were deemed statistically significant and then compared to uncover overlapping ontologies and pathways between studies.

## 3 Results

For studies focusing on folate exposures in relation to DNA methylation, our search strategy yielded 368 publications (flow chart in Supporting Information 3). Based on the predefined exclusion criteria, 364 records were excluded. The remaining four reports were evaluated based on the full‐texts, after which one report was excluded. Three studies investigating the association between serum folate levels or dietary folate intake and DNA methylation were included (F1 [17]; F2 [18]; F3 [19]).

For studies focusing on DNA methylation in relation to chemotherapy‐induced toxicities, the search strategy yielded a total of 29 studies (flow chart in Supporting Information 3). A total of 26 records were excluded based on the predetermined exclusion criteria. The remaining three reports were evaluated based on their full‐text and all three reports were included (T1 [27]; T2 [28]; T3 [29]). In T1, the toxicities hand‐foot syndrome (HFS), thrombocytopenia, anemia, bone marrow suppression, neutropenia, diarrhea, nausea, and vomiting were evaluated, in T2 only HFS and in T3 only thrombocytopenia was evaluated. Studies T1‐T3 are from the same study with potentially overlapping study populations (sample sizes: T1, *n* = 21; T2, *n* = 56; T3, *n* = 56). A total of six publications were included in this study (Table 1). Ethics approval was given within each individual study included in this study.

**TABLE 1:** Overview of the included studies.

Study ID	First author + year	Country	Study type	Study population	No. of participants	Exposure	Outcome	Main findings
Folate exposures								
F1	Kok, 2015 [17]	The Netherlands	Cross‐sectional analyses within a randomized placebo‐controlled trial	Adults with mildly elevated homocysteine levels aged 65–75 years	87	Baseline serum folate levels (measured before the start of intervention)	Genome‐wide DNA methylation in buffy coat (450K array)	Baseline serum folate levels were associated with DNA methylation of 0 DMPs and 173 DMRs (BH‐adjusted *p* value < 0.05)
F2	Mandaviya, 2019 [18]	Europe^a^ and United States	Meta‐analysis of 10 different cohort studies	Adults	5841	Dietary intake of folate in µg/d (FFQ)	Genome‐wide DNA methylation in whole blood or buffy coat (450K array)	Dietary folate intake was associated with DNA methylation of 6 DMPs and 74 DMRs (BH‐adjusted *p* value < 0.05)
F3	Perrier, 2019 [19]	Europe^b^	Cross‐sectional analyses within a nested case‐control study	Postmenopausal women without prevalent cancers at recruitment (except non‐melanoma skin cancer)	450	Dietary intake of folate in µg/day (FFQ or interview methods)	Genome‐wide DNA methylation in buffy coat (450K array)	Dietary folate intake was associated with DNA methylation of 0 DMPs and 24 DMRs (q_DMR_ < 0.05)
Chemotherapy‐induced toxicities								
T1	Li, 2021^c^ [27]	China	Cross‐sectional study	Adults with CRC undergoing capecitabine‐based adjuvant chemotherapy	21	Genome‐wide DNA methylation in malignant colorectal tissue (EPIC array)	Chemotherapy‐induced toxicities^d^ (CTCAE_v4.03)	Numerous genes and DMRs associated with different chemotherapy‐induced toxicities (*p* value ≤ 0.05 and average absolute Δß‐value ≥0.1)^e^
T2	Li, 2021^c^ [28]	China	Cross‐sectional study	Adults with CRC undergoing capecitabine‐based adjuvant chemotherapy	56	Genome‐wide DNA methylation in malignant colorectal tissue (EPIC array)	HFS (CTCAE_v4.0)	Multiple CpGs (*n* = 13 756) linked to HFS (*p* value < 0.05 and ±0.1 fold change)
T3	Yao, 2022^c^ [29]	China	Cross‐sectional study	Adults with CRC undergoing capecitabine‐based adjuvant chemotherapy	56	Genome‐wide DNA methylation in malignant colorectal tissue (EPIC array)	thrombocytopenia (CTCAE_v4.0)	Multiple CpGs (*n* = 5090) linked to thrombocytopenia (log‐fold change values >0 or <0, *p* value <0.05, and absolute Δß‐value >0.01)^e^

Abbreviations: BH, Benjamini Hochberg; CTCAE, Common Terminology Criteria for Adverse Events; DMP, DNA methylated positions; DMR, DNA methylated regions; EPIC, Infinium MethylationEPIC; FFQ, food frequency questionnaire; HFS, hand‐foot syndrome; 450K, Illumina Infinium Human Methylation 450K BeadChip.

^a^ The Netherlands, Italy, Finland, and the United Kingdom.

^b^ Germany, Greece, Italy, the Netherlands, Spain, and the United Kingdom.

^c^ From the same study, potentially overlapping study populations.

^d^ Anemia, bone marrow suppression, diarrhea, hand‐foot syndrome, nausea, vomiting, neutropenia, thrombocytopenia.

^e^ *β* = Intensity [methylated]/Intensity [methylated + unmethylated].

### 3.1 Folate Exposures and DNA Methylation

**FIGURE 1:**
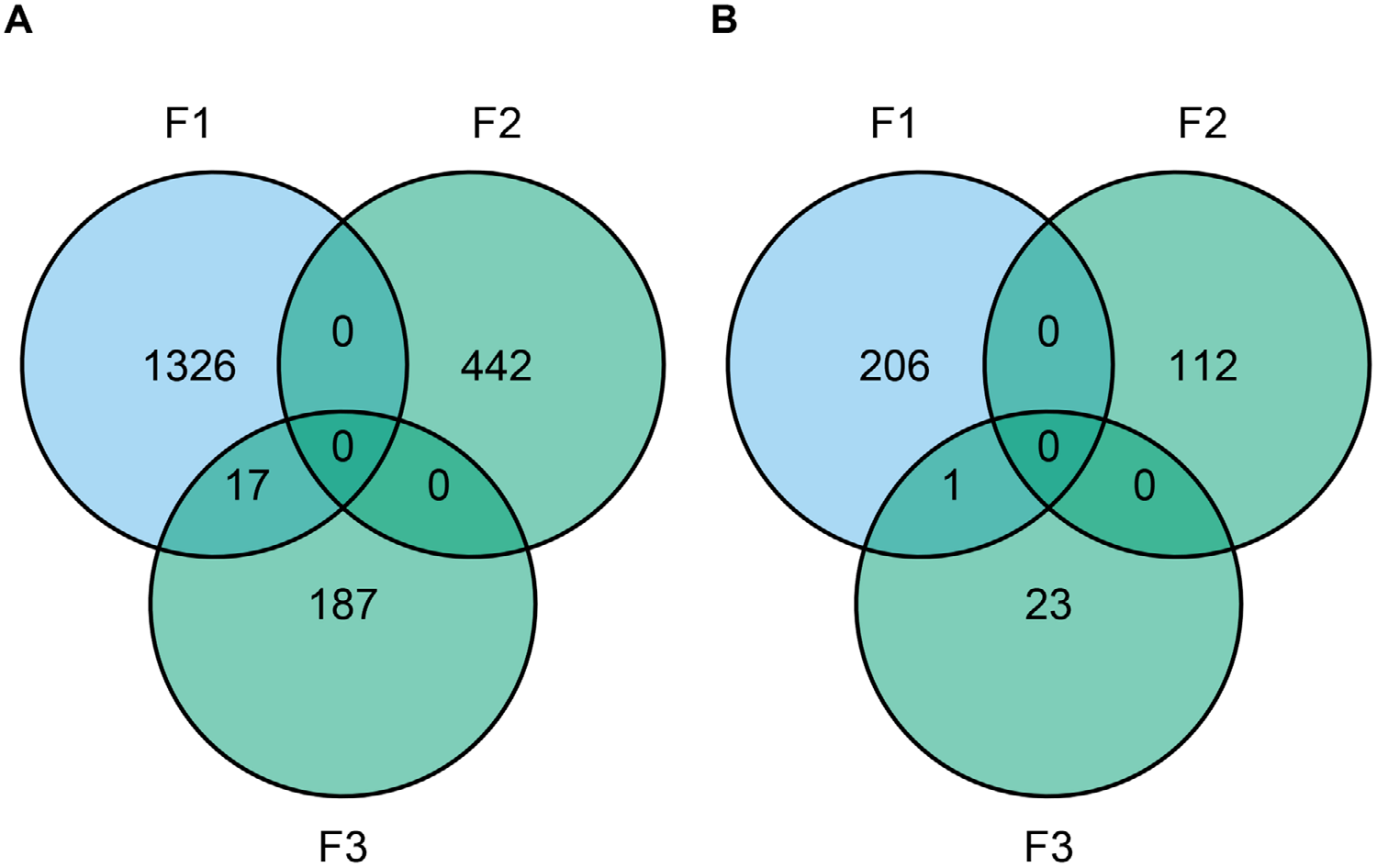
Overlapping methylation profiles between three studies investigating folate‐associated DNA methylation in adults (F1‐3). (A) Overlap for genes that were associated with folate exposures in the three different studies; (B) overlap for CpGs that were associated with folate exposures in the three different studies. Folate exposures were measured as serum levels (blue color) or as dietary intake (green color).

There were 1343 (F1), 442 (F2), and 204 (F3) CpGs identified with folate‐associated methylation. A total of 17 unique CpGs, all situated within *HOXA4*, overlapped between two of the three studies (F1 and F3) (Figure 1), which was unlikely due to chance (*p* < 0.001). When focusing on gene level, there were 207 (F1), 112 (F2), and 24 (F3) genes with folate‐associated DNA methylation patterns identified. Of these, only *HOXA4* overlapped among two studies (F1 and F3), which was likely due to chance (*p* = 0.21).

### 3.2 DNA Methylation and Chemotherapy‐Induced Toxicities

In total, 3680 (T1) and 13 756 (T2) CpGs and 2339 (T1) and 5386 (T2) genes were associated with HFS. There were 53 CpGs (*p* = 0.97) and 949 genes (*p* < 0.001) overlapping between both studies. A total of 2876 (T1) and 5090 (T3) CpGs and 1195 (T1) and 2393 (T3) genes were observed to be associated with thrombocytopenia, of which 13 CpGs (*p* = 1.0) and 272 genes (*p* < 0.001) overlapped. Additionally, there were 205, 88, 7625, 75, 20, and 18 CpGs and 40, 28, 162, 27, 11, and 13 genes associated with anemia, bone marrow suppression, neutropenia, diarrhea, nausea, and vomiting, respectively (T1). As these outcomes were only considered in one publication, no overlap could be calculated.

### 3.3 Folate Exposures, Chemotherapy‐Induced Toxicities, and DNA Methylation

A total of 19 CpGs overlapped between folate‐associated methylation and methylation‐associated HFS across all study comparisons (Table 2 and Supporting Information 4), which were all likely to be chance findings. One CpG site, cg16651126, located within the promoter region of *HOXA4*, overlapped between three studies (F1, T1, and T2). In contrast to the CpGs, there were 118 overlaps for 100 genes observed between folate‐associated methylation and methylation‐associated HFS across all study comparisons (Table 2 and Supporting Information 4). The 24 gene overlaps observed between F2 and T1 were unlikely due to chance (*p* = 0.001) (Table 2); however, all other overlaps between studies were likely to be chance findings. Of note, the *HOXA4* gene overlapped among F1, F3, and T2. DNA Methylation of *HOXA4* increased with higher levels of serum folate in F1 (ß‐coefficient 0.08), while in study F3 *HOXA4* methylation decreased with higher folate intake (ß‐coefficient –0.016). In T2, individuals with HFS compared to those without HFS, had hypermethylation of *HOXA4*. In addition, another HOX gene, *HOXA5*, overlapped between F3 and T2, in which higher folate intake was associated with increased DNA methylation (ß‐coefficient 0.019) (F3) and those with HFS compared to those without HFS had hypermethylation of *HOXA5* (T2).

**TABLE 2:** The overlapping genes and CpGs per study investigating folate‐associated DNA methylation (F1‐3) and per study investigating DNA methylation‐associated toxicities (T1‐3).

		F1		F2		F3	
		No. of overlaps	*p* value^a^	No. of overlaps	*p* value^a^	No. of overlaps	*p* value^a^
HFS (T1)	CpGs	7	0.93	1	0.98	0	—
	Genes	18	0.88	24	**0.001** ^b^	3	0.50
HFS (T2)	CpGs	5	1.00	2	1.00	5	0.78
	Genes	33	1.00	31	0.32	9	0.13
Thrombocytopenia (T1)	CpGs	1	1.00	8	**0.011** ^c^	0	—
	Genes	10	0.73	8	0.30	1	0.75
Thrombocytopenia (T3)	CpGs	2	1.00	2	0.97	1	0.91
	Genes	17	0.94	15	0.28	4	0.28
Neutropenia (T1)	CpGs	7	1.00	8	0.53	0	—
	Genes	1	0.80	3	0.05	0	
Anaemia (T1)	CpGs	0	—	0	—	0	—
	Genes	0	—	0	—	0	—
Bone marrow suppression (T1)	CpGs	0	—	0	—	0	—
	Genes	0	—	0	—	0	—
Diarrhoea (T1)	CpGs	0	—	0	—	0	—
	Genes	1	0.27	0	—	0	—
Nausea (T1)	CpGs	0	—	0	—	0	—
	Genes	0	—	0	—	0	—
Vomiting (T1)	CpGs	0	—	0	—	0	—
	Genes	0	—	0	—	0	—

^a^ *p* value calculated using hypergeometric tests, in bold the significant *p* values (*p* < 0.05).

^b^ *PPT2, PRRT1, NR3C1, SDCCAG8, AKT3, CUX1, PHF12, SEZ6, TMC6, SNCA, KIAA0408, MKL1, SH3PXD2A, EPG5, TMEM33, FBXO31, C10orf54, LSP1, NFIX, GFRA2, KDM2B, NFAM1, SEPT9*, and *SPIDR*.

^c^ cg18082788, cg00073460, cg04275695, cg09678939, cg13136655, cg15132169, cg17501395, and cg18951352.

At total of 14 overlaps for 14 CpGs were observed between folate‐associated methylation and methylation‐associated thrombocytopenia, of which eight CpGs, all situated within *ZC3H12D*, were common between F2 and T1 and were unlikely due to chance (*p* = 0.011) (Table 2, Supporting Information 5). Moreover, 55 overlaps for 50 genes were observed for folate‐associated methylation and methylation‐associated thrombocytopenia across all study comparisons (Table 2, Supporting Information 5). All overlaps between the studies were likely to be chance findings.

Fifteen CpGs and four genes and were common in both folate‐associated methylation and methylation‐associated neutropenia (Table 2, Supporting Information 6), both of which were likely due to chance. While folate‐associated methylation and methylation‐associated diarrhea was observed within the *NUP35* gene, it was not found within specifically overlapping CpGs and was also likely to be due to chance. No overlapping CpGs or genes were observed between folate‐associated and methylation‐associated anemia, bone marrow suppression, vomiting, and nausea (Table 2).

### 3.4 GO and KEGG Pathway Analyses

DNA methylation in response to folate was linked to 20 GO processes in one study (F1), but none in either of the other studies (F2 and F3). HFS, thrombocytopenia, and neutropenia‐associated methylation profiles were linked to 822 (T1) and 989 (T2) (500 in common), 743 (T1) and 445 (T3) (306 in common), and 85 (T3) GO processes, respectively. None of the processes were overlapping between the studies on folate and toxicities.

**FIGURE 2:**
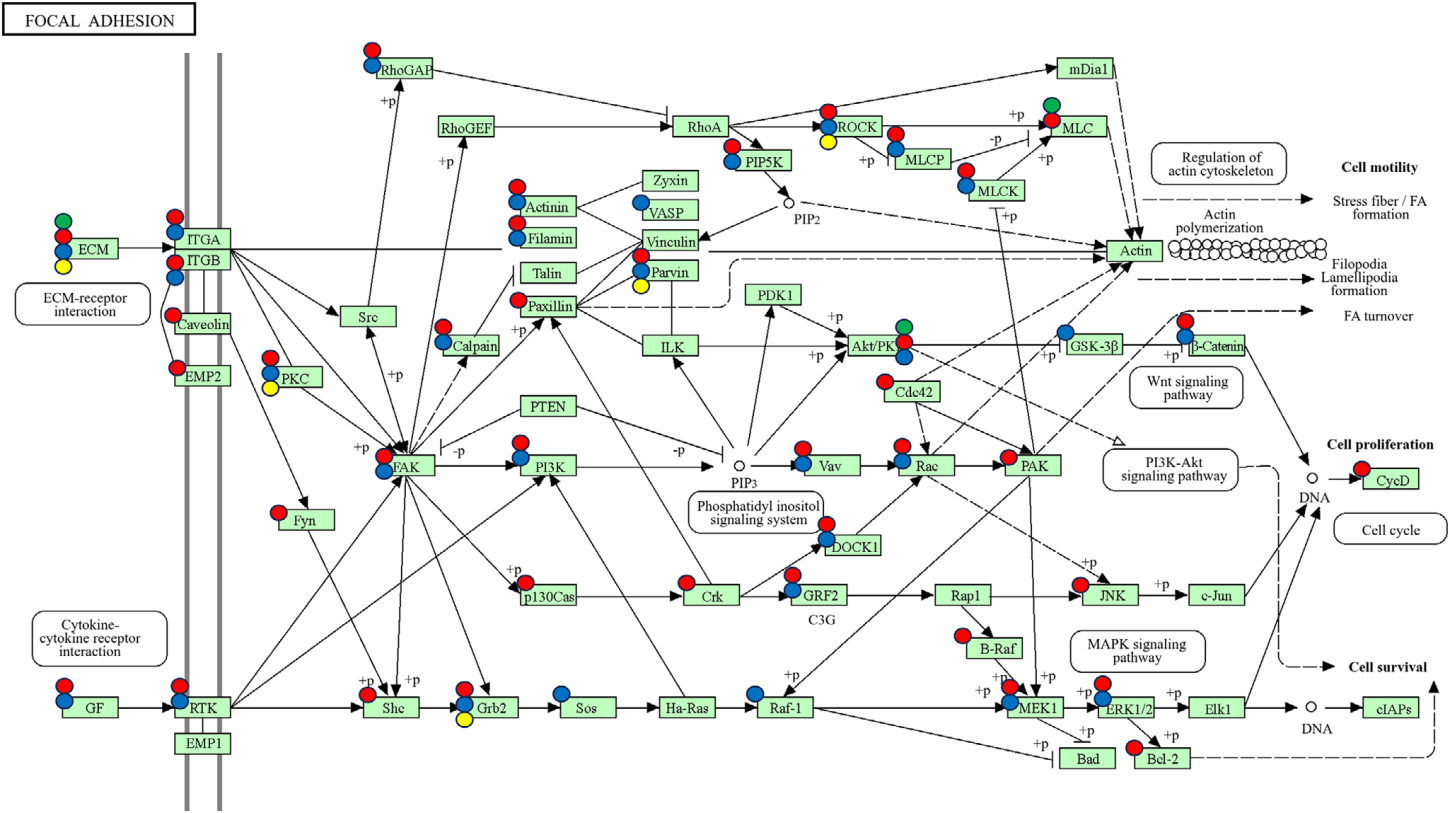
The KEGG focal adhesion pathway. Green dots symbolize genes/gene sets for which folate intake was associated with DNA methylation, red dots represent the genes/gene sets for which DNA methylation was associated with HFS, blue dots for the genes/gene sets for which methylation was associated with thrombocytopenia, and yellow dots represent the genes/gene sets for which methylation was associated with neutropenia. The image is obtained from KEGG, which is developed by Kanehisa Laboratories. [47, 48, 49]

KEGG pathway analysis highlighted 2 (F1), 13 (F2), and 0 (F3) pathways for folate‐associated DNA methylation (0 in common) (Tables 3 and 4). Although just outside the set‐imposed threshold of significance, the KEGG pathway “focal adhesion” was observed in F2 (*p* = 0.055). Meanwhile, 88 (T1) and 115 (T2) pathways for HFS (57 in common), 105 (T1) and 82 (T3) pathways for thrombocytopenia (59 in common), and 17 pathways for neutropenia (T3) were observed. The overlap in highlighted KEGG pathways between HFS (T2), thrombocytopenia (T1), neutropenia (T1), and F2 was unlikely due to chance (*p* < 0.001) (Table 3). In total, there were 37 overlaps of 13 different KEGG pathways common to folate and the different toxicities (Tables 3 and 4). The KEGG pathway “focal adhesion” was common to folate, HFS, thrombocytopenia, and neutropenia (Figure 2).

**TABLE 3:** The number of common KEGG pathways in which methylated gene profiles were related to folate exposures (F1‐F3) and toxicities (T1‐T3). In brackets, the number of highlighted KEGG pathways per individual study.

	F1 [2]	F2^a^ [13]		F3 [0]
	No. of overlaps	No. of overlaps	*p* values^b^	No. of overlaps
HFS (T1) [88]	0	1	0.98	0
HFS (T2) [115]	0	12	**<0.001**	0
Thrombocytopenia (T1) [105]	0	12	**<0.001**	0
Thrombocytopenia (T3) [82]	0	6	0.05	0
Neutropenia (T1) [17]	0	6	**<0.001**	0

^a^ Including the KEGG pathway “focal adhesion.”

^b^ *p* value calculated using hypergeometric tests, in bold the significant *p* values (*p* < 0.05).

**TABLE 4:** KEGG pathway in which folate exposures were associated with DNA methylation presented for each of the studies separately.

KEGG ID	KEGG pathway	*p* value	Genes involved	Common KEGG pathways with T1‐3
F1				
hsa03010	Ribosome	0.008	*RPL4, RPL3, MRPL18, RPL34, MRPS21, RPS27A*	—
hsa03040	Spliceosome	0.023	*SNRNP40, PRPF38A, RBM8A, PRPF3, HNRNPA1, RBM22*	—
F2				
hsa04550	Signalling pathways regulating pluripotency of stem cells	0.003	*WNT6, WNT10A, ID2, AKT3, WNT9B*	Thrombocytopenia (T1)
hsa05225	Hepatocellular carcinoma	0.006	*WNT6, WNT10A, AKT3, WNT9B, ARID1B*	HFS (T2), thrombocytopenia (T1), neutropenia (T1)
hsa04916	Melanogenesis	0.009	*POMC, WNT6, WNT10A, WNT9B*	HFS (T2), thrombocytopenia (T1 and T3)
hsa05205	Proteoglycans in cancer	0.011	*WNT6, WNT10A, AKT3, WNT9B, NUDT16L1*	HFS (T2), thrombocytopenia (T1), neutropenia (T1)
hsa05165	Human papillomavirus infection	0.013	*WNT6, WNT10A, AKT3, LAMA3, WNT9B, COL9A3*	HFS (T2), thrombocytopenia (T1 and T3), neutropenia (T1)
hsa05010	Alzheimer disease	0.023	*DKK4, WNT6, WNT10A, AKT3, WNT9B, SNCA*	—
hsa05224	Breast cancer	0.025	*WNT6, WNT10A, AKT3, WNT9B*	HFS (T2), thrombocytopenia (T1)
hsa05226	Gastric cancer	0.025	*WNT6, WNT10A, AKT3, WNT9B*	HFS (T2), thrombocytopenia (T1)
hsa04934	Cushing syndrome	0.028	*POMC, WNT6, WNT10A, WNT9B*	HFS (T2), thrombocytopenia (T1 and T3)
hsa04150	mTOR signaling pathway	0.029	*WNT6, WNT10A, AKT3, WNT9B*	HFS (T2), thrombocytopenia (T1), neutropenia (T1)
hsa04390	Hippo signaling pathway	0.029	*WNT6, WNT10A, ID2, WNT9B*	HFS (T2), thrombocytopenia (T1 and T3)
hsa05217	Basal cell carcinoma	0.030	*WNT6, WNT10A, WNT9B*	HFS (T2)
hsa04310	Wnt signaling pathway	0.038	*DKK4, WNT6, WNT10A, WNT9B*	HFS (T2), thrombocytopenia (T1 and T3), neutropenia (T1)
hsa04510	Focal adhesion	0.055	*AKT3, LAMA3, MYL10, COL9A3*	HFS (T1 and T2), thrombocytopenia (T1 and T3), neutropenia (T1)

## 4 Discussion

In this study, we aimed to explore the potential role of folate‐mediated DNA methylation in the context of chemotherapy‐induced toxicities in patients with CRC receiving fluoropyrimidine‐based therapy using a meet‐in‐the‐middle approach. A total of six studies were included with either data on folate‐associated DNA methylation or DNA methylation patterns associated with chemotherapy‐induced toxicities. We observed a significant number of overlapping genes with methylation patterns related to both folate and HFS, and CpGs with methylation related to folate and thrombocytopenia. The KEGG pathway “focal adhesion” was highlighted as potentially relevant, with methylation status being associated with folate, HFS, thrombocytopenia, and neutropenia.

To the best of our knowledge, this is the first study exploring the role of folate‐mediated DNA methylation in fluoropyrimidine‐induced toxicities. There was, however, surprisingly limited overlap of methylated genes and CpGs between the three studies investigating the association between folate exposures and DNA methylation. Only methylation of the *HOXA4* gene, and specifically the CpG site cg16651126 situated within *HOXA4*, was observed in two of the three included studies (F1, F3) [17, 19]. The limited overlap might be explained by the different study designs and populations. Only the study of Kok et al. (F1) [17] measured folate as serum folate; the other two studies (F2, F3) [18, 19] evaluated dietary folate intake. Folate intake is not always strongly associated with circulating folate status [30]. Moreover, the study populations themselves varied, with Perrier et al. (F3) [19] including only postmenopausal women from Europe, Kok et al. (F1) [17] including elderly adults from the Netherlands, and Mandaviya et al. (F2) [18] including adults from different countries in Europe and from the United States. Not only is DNA methylation associated with age [31], but also associated with different lifestyle factors and ethnicity [32, 33, 34, 35, 36], which may have impacted the findings, potentially explaining the variability in the findings between studies. This difference in study designs and populations, therefore, highlights a gap in our understanding of how exposures, specifically folate intake and status, influence the epigenome across populations, and emphasizes the need for further in‐depth investigations.

Nevertheless, we observed genes and CpGs that were linked to methylation profiles associated with both folate and certain chemotherapy‐induced toxicities. We mainly observed a potential relation between folate‐mediated DNA methylation and HFS and thrombocytopenia. The methylation pattern overlap of the toxicities with the folate studies was only observed in T1 and not T2 or T3, potentially due to a different study population. However, the observed overlap in DNA methylation patterns between folate and HFS and thrombocytopenia, but not other toxicities, may also suggest that for some toxicities folate‐associated methylation may play a role while for others it might not. Noteworthy, two *HOX* genes, *HOXA4* and *HOXA5*, were identified in both folate‐associated methylation and methylation‐associated HFS. *HOX* genes and the DNA methylation of these genes are important in developmental processes and have been linked to health and disease, including neural tube defects (NTDs) [37] for which folic acid supplementation during early pregnancy reduces the risk [38]. Also, higher expression of *HOXA4* was associated with a shorter progression‐free survival in 71 patients with ovarian cancer undergoing adjuvant platinum‐based chemotherapy (HR 1.20, 95% CI 1.07–1.34) [39]. Overexpression of *HOXA4* was suggested to enhance resistance to multiple platinum compounds and potentially reduce the production of reactive oxygen species (ROS) in ovarian cancer cell models [39]. The combination of capecitabine and oxaliplatin, a platinum compound, was administered in the studies included in this study [27, 28, 29], and therefore might explain the observed association between *HOXA4* methylation and HFS. Overall, the role of *HOXA4* methylation and expression in capecitabine and oxaliplatin treatment tolerance is an area for further research.

The pathway analyses suggested genes involved in multiple cancer‐related pathways, such as “gastric cancer,” “mTOR signaling pathway,” and “breast cancer,” had methylation profiles related to both folate and toxicities. As the study population of the toxicity studies (T1‐T3) comprised of individuals diagnosed with CRC, and folate has been linked to cancer and specifically CRC before [40, 41], these findings might be more related to the disease itself and less so with the experienced toxicities. Nevertheless, methylation of genes in the “focal adhesion” pathway were related to folate and multiple toxicities, namely HFS, thrombocytopenia, and neutropenia. The KEGG pathway “focal adhesion” was also highlighted previously in a study seeking to determine tumor microenvironment‐related genes linked to 5‐FU resistance among patients with CRC [42]. Preclinical studies observed that silencing the focal adhesion kinase (*FAK*) gene may potentiate cytotoxicity of 5‐FU both in vitro and in vivo [43, 44], the authors suggested that these affects might be through the suppression of the AKT/NF‐κB signaling pathway [43, 44]. Altogether, identifying folate‐associated methylation of genes within the “focal adhesion” pathway might help us understand the mechanism linking folate exposures and chemotherapy‐induced toxicities; however, based on the current evidence, we cannot confirm this relationship yet.

This study has some limitations. First, only six studies met the inclusion criteria and could therefore be included in this study. The studies investigating folate‐associated DNA methylation originated from Europe and the United States (F1‐F3) [17, 18, 19], while the studies investigating DNA methylation and toxicities were conducted in China (T1‐T3) [27, 28, 29]. This could have resulted in a few overlapping genes and CpGs being identified, as epigenetic profiles are shown to be depending on lifestyle factors and ethnicity [32, 33, 34]. Moreover, the folate studies all measured DNA methylation in blood, while the toxicities studies measured DNA methylation in malignant tissue, which may have impacted methylation patterns [45, 46]. Second, not all studies provided data on the direction of methylation (hyper‐ or hypo‐methylation) per CpG site in relation to folate exposures or in the context of toxicities. Hyper‐ or hypo‐methylation is generally linked to either inhibition or activation of the genes, respectively [15], and may influence how we think folate may affect the outcome. Third, we need to elucidate folate‐methylation and methylation‐toxicity relationships before we can have better insights in the potential role of folate‐mediated DNA methylation in toxicities, as we observed limited overlap across studies investigating folate exposures or toxicities. We especially expected more overlap between the toxicity studies as these study populations were similar. Nevertheless, to the best of our knowledge, this is the first study exploring the potential role of folate‐mediated DNA methylation in the context of chemotherapy‐induced toxicities. We were able to observe common genes and CpGs, such as *HOXA4*, and potentially important pathways, such as “focal adhesion,” associated with folate‐associated methylation and methylation‐associated toxicities. This study therefore provides a first indication that folate‐mediated DNA methylation may play a role in certain chemotherapy‐induced toxicities, such as HFS and thrombocytopenia.

Overall, this study aimed to explore the potential role of folate‐mediated DNA methylation in chemotherapy‐induced toxicities. We observed some overlap between DNA methylation profiles related to both folate exposures and certain chemotherapy‐induced toxicities, mainly HFS and thrombocytopenia, suggesting that folate‐mediated methylation may be important in understanding the role of folate in toxicities. Future studies are warranted to investigate folate‐mediated methylation, to ultimately provide solid evidence on the role of folate‐mediated methylation in toxicities.

## Conflicts of Interest

The authors declare no conflicts of interest.

## Peer Review

The peer review history for this article is available at https://publons.com/publon/10.1002/mnfr.70127.

## Supporting information



Supporting information

Supporting information

## Data Availability

Data can be shared on request, which can be sent to Nienke Zwart, MSc, Division of Human Nutrition and Health, Wageningen University & Research, The Netherlands (e‐mail: nienke.zwart@wur.nl).
