# Supplementary material for: Folate‐Associated DNA Methylation and Chemotherapy‐Induced Toxicities in Patients With Colorectal Cancer
Source: Mol Nutr Food Res. 2025 May 27;69(14):e70127. doi: 10.1002/mnfr.70127 (PMC12280844; doi:10.1002/mnfr.70127)
Supplement: Supplementary file 1 — Supporting information [file MNFR-69-e70127-s002.docx]

**Supplementary File 1.** The search strings used to identify papers

| Search strategy | Search query |
| --- | --- |
| 1 | (“adults” [MeSH] OR “Middle Aged” [MeSH] OR “Aged” [MeSH] OR “adult*” [Title/Abstract] OR “elder*” [Title/Abstract]) AND (“folic acid” [MeSH] OR “folic acid” [Title/Abstract] OR "folate" [Title/Abstract] OR "vitamin B9"[Title/Abstract] OR "vitamin B11"[Title/Abstract]) AND (“DNA methylation” [MeSH] OR “DNA methylation” [Title/Abstract]) |
| 2 | (“DNA methylation” [MeSH] OR “DNA methylation” [Title/Abstract])  AND (“Colorectal Neoplasms” [MeSH] OR “colorectal*” [Title/abstract]) AND  ("fluoropyrimidine*"[Title/Abstract] OR "5-FU"[Title/Abstract] OR "5FU"[Title/Abstract] OR "fluorouracil"[Title/Abstract] OR "capecitabine"[Title/Abstract] OR "CAPOX"[Title/Abstract] OR "XELODA"[Title/Abstract] OR "folf*"[Title/Abstract]) AND ("Drug-Related Side Effects and Adverse Reactions"[MeSH Terms] OR "Treatment outcome"[MeSH Terms] OR "toxic*"[Title/Abstract] OR "side effect*"[Title/Abstract] OR "side effect*"[Title/Abstract] OR "complication*"[Title/Abstract] OR "tolerance"[Title/Abstract] OR "adverse effect*"[Title/Abstract] OR "adverse effect*"[Title/Abstract] OR "adverse event*"[Title/Abstract] OR "adverse drug reaction*"[Title/Abstract])) |

**Supplementary File 2.** The redundant extracted CpGs from the DMRs.

| Study | Percentage of redundant CpGs |
| --- | --- |
| Kok et al. (F1) | 0% |
| Mandaviya et al. (F2) | 2.8% |
| Perrier et al. (F3) | 7.4% |


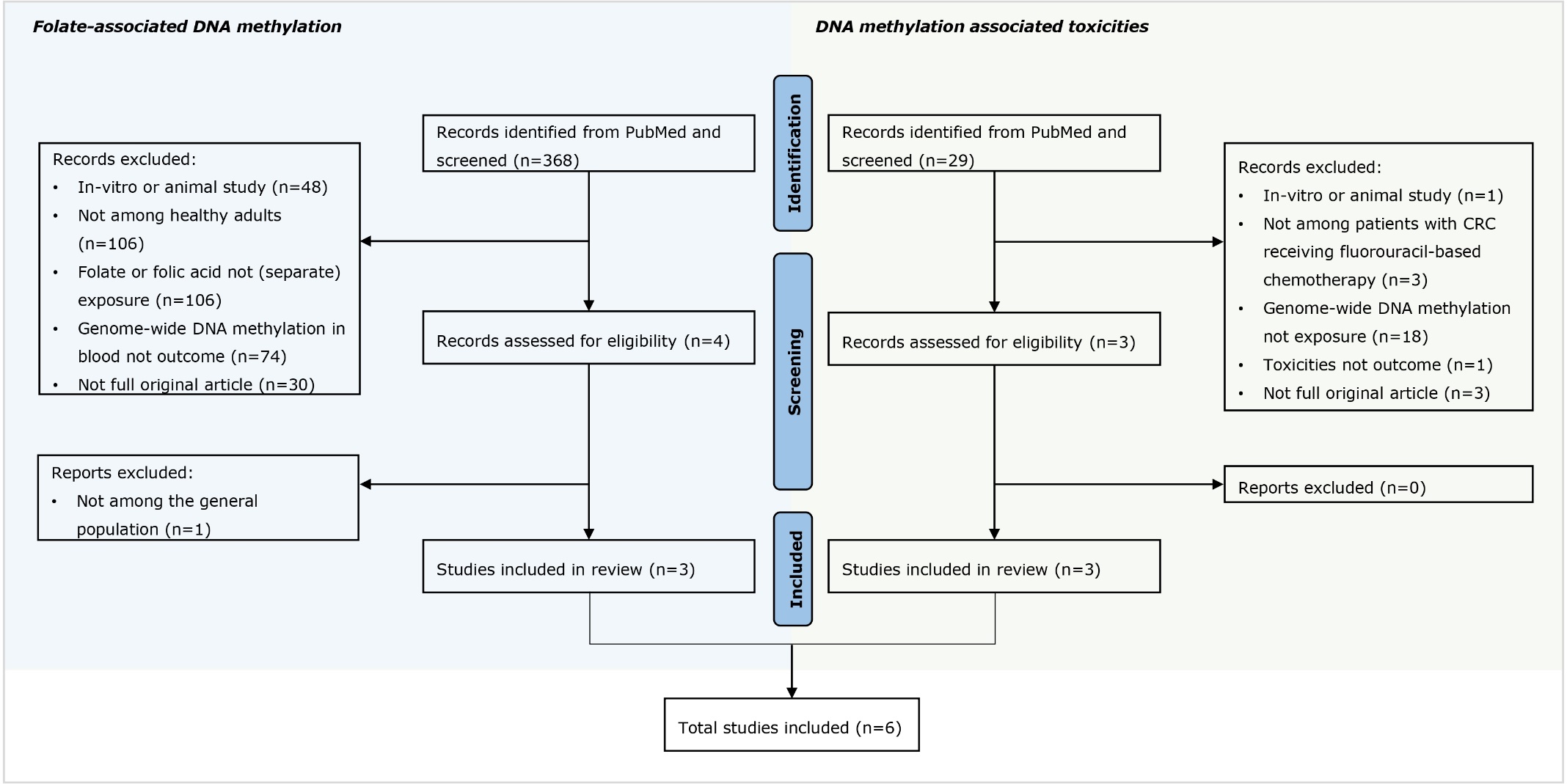


**Supplementary File 3.** Flow chart of the study.
